# Patient-centered primary care and self-rated health in 6 Latin American and Caribbean countries: Analysis of a public opinion cross-sectional survey

**DOI:** 10.1371/journal.pmed.1002673

**Published:** 2018-10-09

**Authors:** Frederico Guanais, Svetlana V. Doubova, Hannah H. Leslie, Ricardo Perez-Cuevas, Ezequiel García-Elorrio, Margaret E. Kruk

**Affiliations:** 1 Social Protection and Health Division, Inter-American Development Bank, Lima, Peru; 2 Epidemiology and Health Services Research Unit, Mexican Institute of Social Security, Mexico City, Mexico; 3 Department of Global Health and Population, Harvard T.H. Chan School of Public Health, Boston, Massachusetts, United States of America; 4 Center for Health Systems Research, National Institute of Public Health, Cuernavaca, Mexico; 5 Institute for Clinical and Health Effectiveness, Buenos Aires, Argentina; Edinburgh University, UNITED KINGDOM

## Abstract

**Background:**

Despite the substantial attention to primary care (PC), few studies have addressed the relationship between patients’ experience with PC and their health status in low-and middle-income countries. This study aimed to (1) test the association between overall patient-centered PC experience (OPCE) and self-rated health (SRH) and (2) identify specific features of patient-centered PC associated with better SRH (i.e., excellent or very good SRH) in 6 Latin American and Caribbean countries.

**Methods and findings:**

We conducted a secondary analysis of a 2013 public opinion cross-sectional survey on perceptions and experiences with healthcare systems in Brazil, Colombia, El Salvador, Jamaica, Mexico, and Panama; the data were nationally representative for urban populations. We analyzed 9 features of patient-centered PC. We calculated OPCE score as the arithmetic mean of the PC features. OPCE score ranged from 0 to 1, where 0 meant that the participant did not have any of the 9 patient-centered PC experiences, while 1 meant that he/she reported having all these experiences. After testing for interaction on the additive scale, we analyzed countries pooled for aim 1, with an interaction term for Mexico, and each country separately for aim 2. We used multiple Poisson regression models double-weighted by survey and inverse probability weights to deal with the survey design and missing data. The study included 6,100 participants. The percentage of participants with excellent or very good SRH ranged from 29.5% in Mexico to 52.4% in Jamaica. OPCE was associated with reporting excellent or very good SRH in all countries: adjusting for socio-demographic and health covariates, patients with an OPCE score of 1 in Brazil, Colombia, El Salvador, Jamaica, and Panama were more likely to report excellent or very good SRH than those with a score of 0 (adjusted prevalence ratio [aPR] 1.61, 95% CI 1.37–1.90, *p <* 0.001); in Mexico, this association was even stronger (aPR 4.27, 95% CI 2.34–7.81, *p <* 0.001). The specific features of patient-centered PC associated with better SRH differed by country. The perception that PC providers solve most health problems was associated with excellent or very good SRH in Colombia (aPR 1.38, 95% CI 1.01–1.91, *p =* 0.046) and Jamaica (aPR 1.21, 95% CI 1.02–1.43, *p =* 0.030). Having a provider who knows relevant medical history was positively associated with better SRH in Mexico (aPR 1.47, 95% CI 1.03–2.12, *p =* 0.036) but was negatively associated with better SRH in Brazil (aPR 0.71, 95% CI 0.56–0.89, *p =* 0.003). Finally, easy contact with PC facility (Mexico: aPR 1.35, 95% CI 1.04–1.74, *p =* 0.023), coordination of care (Mexico: aPR 1.53, 95% CI 1.19–1.98, *p =* 0.001), and opportunity to ask questions (Brazil: aPR 1.42, 95% CI 1.11–1.83, *p =* 0.006) were each associated with better SRH. The main study limitation consists in the analysis being of cross-sectional data, which does not allow making causal inferences or identifying the direction of the association between the variables.

**Conclusions:**

Overall, a higher OPCE score was associated with better SRH in these 6 Latin American and Caribbean countries; associations between specific characteristics of patient-centered PC and SRH differed by country. The findings underscore the importance of high-quality, patient-centered PC as a path to improved population health.

## Abstract 2

Svetlana Doubova and colleagues investigate which elements of patient-centered primary care are associated with better individual self-rated health in Brazil, Colombia, El Salvador, Jamaica, Mexico, and Panama.

## Author summary

**Why was this study done?:**

- In the 40 years since the Declaration of Alma-Ata, empirical studies using ecological data have shown positive effects of access to primary care on population health outcomes, such as child mortality, adult overall mortality, and adult avoidable hospitalizations, both in high-income countries and in low- and middle-income countries (LMICs).
- In the context of LMICs, the centrality of primary care has been questioned by findings of its poor quality and its limitations in adapting to urbanization and to the epidemiological transition, as well as the increasing population demand for responsive, high-quality services.
- To our knowledge, no individual-level studies have examined the relationship between the attributes of patient-centered primary care and self-rated health (SRH) in the context of LMICs.

**What did the researchers do and find?:**

- Using person-level survey data representative of the urban population of 6 countries in Latin America and the Caribbean, the current study shows that individuals who reported receiving more patient-centered primary care overall were more likely to report excellent or very good SRH status in all countries. Features of primary care associated with better SRH differed between countries.

**What do these findings mean?:**

- The expansion of primary care in LMICs can be informed by better evidence on which of its features—including ease of communication, comprehensiveness of care, and support for coordination of care—are associated with better self-reported health status.
- While individual-level administrative data are not yet widely available for health systems in LMICs, patient-reported survey data may serve as an instrument to assess healthcare services and to inform policy-makers in their efforts to increase the quality of primary care services.

## Introduction

Primary care (PC) has been described as being uniquely positioned to promote health and well-being at the population level [1–3]. Its central role in providing adequate, efficient, and equitable access to preventive and curative healthcare was strongly emphasized by the Declaration of Alma-Ata 40 years ago [2]. However, PC’s centrality has been questioned by findings of its poor quality, especially in low- and middle-income countries (LMICs), and its limitations in adapting to urbanization and the epidemiological transition, as well as increasing population demand for responsive, high-quality services [4–6].

More recently, patient-centered healthcare has emerged as a person-oriented model of care aimed at meeting population needs, expectations, and preferences. Studies from the United States and the United Kingdom have shown the positive effect of patient-centered healthcare in improving the quality of the processes of care, reducing hospitalizations and emergency visits (and consequently healthcare costs), and improving users’ satisfaction and self-management [7–9]. Within PC, a number of patient-centered healthcare attributes have been shown to be associated with perception of good healthcare quality, such as the availability of a PC provider who “knows relevant information about a patient’s medical history,” “solves most of the health problems,” “spends enough time with the patient,” “coordinates healthcare,” and “is easy to communicate with” [10].

Previous work using ecological data has shown positive effects of PC on population health outcomes, such as child mortality, and avoidable hospitalizations, both in high-income countries and in LMICs [11,12]. However, despite the substantial attention and policy emphasis on PC, few studies have addressed the relationship between patient experience with PC and health in LMICs; none to our knowledge have done so with a cross-country perspective [13,14].

Self-rated health (SRH) is a broadly used measure: individuals evaluate their health status through a Likert scale or compare their health status with individuals of the same age [15]. Though SRH is a subjective indicator of health status, it has been found to be a robust predictor of mortality [15,16]; also, low SRH is associated with increased hospitalization and outpatient care in elderly populations [17]. Research studies addressing the relationship between health service characteristics and SRH have reported that in the US, individuals living in states with a higher ratio of PC physicians to population were more likely to report good SRH than those with a lower ratio of PC physicians [18]. Enhanced accessibility and continuity of PC in the US [19,20] and high total PC quality scores in South Korea were associated with better SRH of health service users [21].

The objectives of the present study were (1) to test the association between overall patient-centered PC experience (OPCE) and SRH in 6 Latin American and Caribbean (LAC) countries and (2) to identify specific features of patient-centered PC associated with better SRH. This work can help inform financing and policies at a moment of renewed global attention to PC.

## Methods

We performed a secondary analysis of a recent (2013) public opinion survey focusing on perceptions and experiences with healthcare systems in 6 LAC countries: Brazil, Colombia, El Salvador, Jamaica, Mexico, and Panama. The detailed methodology of this survey was previously reported elsewhere [10,22,23]. In each country, the survey included a nationally representative urban sample of the population that comprised between 1,500 and 1,506 adults per country. According to the 2017 revision of *World Population Prospects* [24], the urban population constitutes the majority in these countries, ranging from 54% in Jamaica to 85% in Brazil. In total, 330 million individuals reside in urban areas in these countries.

During 2012 and 2013, Harris Interactive collected the data through telephone interviews. The sample frame for the survey consisted of random digit dialing listings of landline and mobile phone numbers in each country. The survey used an adapted version of the methodology and questionnaire that the Commonwealth Fund has been applying in Europe, Australia, Canada, and the US over the past 15 years [25]. The selection criteria considered any household member aged 18+ years. Only 1 adult per household was interviewed.

### Analysis plan

We did not have a formal prospective analysis plan. Prior to seeing the data, we identified the public opinion survey as a unique resource to test associations of healthcare quality and SRH in a representative, multi-country sample. We then reviewed the literature on patient-centered PC and identified 5 key domains (contact with clinic, time spent with provider, patient–provider communication, technical quality and solving problems, and healthcare coordination) relevant for patient-centered healthcare [10,23,25,26]; we mapped items from the survey to these domains and created single-item summaries as well as an overall score. We defined covariates based on relevance to health status and healthcare utilization. We planned to assess all countries in a pooled sample; on identifying substantial variation in the level of SRH between countries, we tested for interaction between patient-centered PC variables and country on the additive scale and report stratified models where evidence of interaction was found.

### Study variables

The dependent variable was “excellent or very good SRH,” obtained from the general SRH report and categorized as 1 = “excellent” or “very good” and 0 = “good,” “fair,” “poor,” or “not sure”.

The survey specified that PC is care provided by the doctors or other health professionals (i.e., nurses, social workers) at the family doctor’s practice or clinic. We selected items related to PC that fall into the domains of patient-centered healthcare identified in the literature [10,23,25,26] and organized them by domain:

- Contact with PC clinic
  - PC facility is easy to contact by telephone during regular office hours
- Time spent with provider
  - PC provider spends enough time with patient
- Patient–provider communication
  - PC provider gives the patient an opportunity to ask questions
  - PC provider explains things in a way that is easy to understand
- Technical quality and solving problems
  - PC provider knows relevant information about the patient’s medical history
  - PC provider advises about healthy lifestyles (healthy food, regular physical activity, and possible stressors)
  - Preventive exams are up to date
  - PC provider solves most of the patient’s health problems
- Healthcare coordination
  - PC provider helps to coordinate care with other physicians or sources of care

The variable “preventive exams up to date” was defined as “yes” when the respondent reported having blood pressure measurement in the last year, serum cholesterol in the last 5 years, and, for women over 40 years, cervical cytology (Pap test) and mammography in the last 3 years.

All other PC variables were measured on a 5-options Likert scale and categorized as 1 = yes (“always” or “often”) and 0 = no (“sometimes,” “rarely or never,” and “not sure”). The decision to categorize the variables this way was based on previous studies [10,23,25,26]. We calculated OPCE as the arithmetic mean of these items, following the recommendation of previous research on the use of patient experience surveys to assess service provision [27]. OPCE score ranged from 0 to 1, where 0 meant that participant did not have any of the 9 patient-centered PC experiences, while 1 meant that he/she reported having all these experiences. We assumed that each component of OPCE score contributed equally to patients’ experiences and that a difference in patient experiences had a constant effect on SRH. We maintained the 9 binary items as individual components of patient-centered PC.

Several socio-demographic and health-service-related factors are associated with poor SRH. Individual factors linked to lower health status include unhealthy lifestyle [28–30] and chronic diseases that affect mental and physical health [31–34]. Although some aspects of the relationship between socio-demographic factors and SRH are still inconclusive, it has been reported that older age, low schooling, low socio-economic status, low social capital, and low health insurance (HI) coverage are associated with poor SRH [35–37].

Based on survey data availability, we included the following covariates: sex, age, education, chronic disease, and the type of HI. The variable education defines the level of education for participants who answered the survey in all countries except El Salvador, where it describes the education of the head of the household. We identified the participant as having a chronic disease if he/she reported that a doctor had told him/her of having arthritis, asthma or chronic lung disease, cancer, diabetes, heart disease, hypertension, or depression. The type of HI was categorized as: government HI (publicly subsidized insurance not related to job affiliation), social security HI (contributory insurance related to job affiliation), and private HI (voluntary private insurance; also, in Brazil and Jamaica, those who reported having private HI provided by workplace). Furthermore, respondents reporting both government and social-security-based HI (4.1% of participants in Colombia and 21.5% in Mexico) were grouped under social security HI. Participants who reported not having HI were placed in the government HI group, because, in all these countries, government HI is freely available for those without social security or private HI.

### Statistical analysis

We used descriptive statistics to analyze the characteristics and PC experiences of the study participants. We performed a bivariable analysis including chi-squared tests between the dependent variable (SRH) and each independent variable (PC experience) or categorical covariate. We used Student *t* tests for comparison of the continuous variable OPCE score between people who reported excellent or very good SRH and those who reported good, fair, or poor (or not sure) SRH.

The survey asked the complete set of questions about PC experiences only to respondents who affirmed “having a regular doctor or regular place for primary care.” This skip pattern results in a high percentage of missing data, given that lack of access to a regular source of PC ranged from 16.3% in Jamaica to 43.1% in El Salvador; in addition, several PC variables also had missing information (S1 Table). In sum, in the 6 countries there were 6,100 participants with complete information, which represented 67.7% of the initial sample of 9,012. Thus, we applied a double-weighted strategy with the use of survey weights to account for the survey sample design and stabilized inverse probability (IP) weights to correct for potential selection bias [38]. This technique consists in assigning a weight to individuals with complete information so that they account for themselves as well as for those with similar characteristics who had missing information. It assumes that those with missing information are similar to those with complete information who share the same measured covariates [38]. In particular, to apply this technique to adjust for the missingness induced by not having a regular PC clinic or doctor, we assumed that the PC experience of individuals without a regular PC clinic or doctor can be represented by those with a regular PC clinic or doctor conditional on the specified covariates, i.e., that there are no unmeasured confounders that are a common cause of both having access to a regular PC clinic or doctor and SRH. We first compared the number of PC visits between those with and without a regular PC clinic or doctor. We found that the mean number of visits in the last 12 months in the group with a regular doctor was 3.07 and in the group without a regular doctor was 2.09 (*p <* 0.001). We then generated the denominator and numerator of the IP weights. The denominator for stabilized IP weights was the probability of having missing data conditional on the following covariates: sex, age, education, type of HI, and presence of chronic disease. The numerator was the probability of having missing data regardless of the covariates.

We used Poisson regression models with robust variance as recommended for cross-sectional studies with high-prevalence binary outcomes [39]. We initially fit pooled models across all 6 countries, and then calculated the relative excess risk due to interaction (RERI) between each country and PC experience as a measure of interaction on the additive scale [40]; additive interaction is more indicative of underlying causal interaction than interaction on the relative (ratio) scale. Where evidence of interaction was identified (RERI significant at *p* ≤ 0.05), we included interaction terms for country in the pooled multiple regression model, or stratified the model by country in the case of multiple interactions identified. Each multiple Poisson regression model included the dependent variable, independent variables, and conceptually relevant covariates. The pooled model included fixed effects for countries to control for country-level heterogeneity and to focus on the effect of the individual-level predictors [41,42].

Finally, we performed a sensitivity analysis in which the IP weights were calculated after the individuals without a regular PC clinic or doctor were dropped. The results were similar to those of the main analysis, suggesting that our findings were not distorted by including everyone when calculating the IP weights. All analyses were performed using the software Stata 14 and considering estimates with *p* ≤ 0.05 to be statistically significant.

### Ethical considerations

The study consists of a secondary data analysis of a public opinion survey focusing on perceptions and experiences with healthcare systems in 6 LAC countries. The survey was commissioned by the Inter-American Development Bank, and the contracted surveying firm was responsible for obtaining all necessary regulatory approvals and verifying compliance with the ethical standards of the ICC/ESOMAR Code on Market, Opinion and Social Research. The survey data for this secondary data analysis were made available by, and their use approved by, the Inter-American Development Bank.

## Results

Tables 1 and 2 present the characteristics of study participants from 6 LAC countries (*n =* 6,100) double-weighted by survey and stabilized IP weights. Slightly more women than men participated in all countries (52.2% versus 47.8% in the full sample). Participants reported lower education levels in Brazil, Panama, and El Salvador (62.7%, 37.5%, and 33.4% with elementary school or less, respectively), while approximately half the sample had completed secondary school in Mexico, Colombia, and Jamaica. Government HI predominated in Brazil and Jamaica (76.5% and 61.5%, respectively), while social security HI was more common in Colombia, Panama, Mexico, and El Salvador (65.1%, 62.4%, 48.9%, and 47.4%, respectively). The proportion with private HI ranged from 9.8% in Colombia and El Salvador to 38.5% in Jamaica. Report prevalence of chronic conditions ranged from 31.5% in El Salvador to 52.2% in Jamaica. Finally, the percentage of participants with excellent or very good SRH was highest in Jamaica (52.4%), declining to a low of 29.5% in Mexico.

**Table 1:** Characteristics of study population according to SRH status: 6-country sample, Brazil, Colombia, and El Salvador.

Characteristic	6-country sample				Brazil			Colombia			El Salvador		
	Total	Excellent or very good SRH	Good, fair, or poor SRH	*p*-Value	Total	Excellent or very good SRH	*p*-Value	Total	Excellent or very good SRH	*p*-Value	Total	Excellent or very good SRH	*p*-Value
**Number of observations**	6,100	2,651	3,449		874	413		1,009	407		812	364	
**Weighted population**	6,007	2,516	3,491		857	399		1,000	404		777	346	
**Variables (weighted percent)**													
**Sex**													
Female	52.2	37.9	62.1	<0.001	50.4	44.3	0.280	51.6	33.3	0.001	54.6	42.1	0.187
Male	47.8	46.2	53.8		49.6	48.9		48.4	47.9		45.4	47.6	
**Age (years)**													
20–25	21.8	51.9	48.1	<0.001	21.1	59.3	<0.001	18.9	42.4	0.569	26.4	52.9	0.063
26–45	43.9	43.3	56.7		43.9	58.8		47.7	40.6		41.6	39.4	
46–59	20.4	37.2	62.8		19.9	28.3		21.8	42.6		18.0	45.7	
≥60	13.9	28.4	71.6		15.1	17.5		11.6	31.9		14.0	42.8	
**Schooling**													
Elementary school or less	34.4	37.0	63.0	<0.001	62.7	44.3	0.325	26.4	34.7	0.255	33.4	43.8	0.033
Secondary school	39.8	42.8	57.2		26.4	49.2		51.0	41.4		25.2	45.5	
College	18.9	48.4	51.6		10.7	53.5		21.4	46.0		20.7	54.0	
Not specified	6.9	43.5	56.5		0.2	63.3		1.2	21.8		20.7	35.2	
**Health insurance**													
Government	41.7	41.8	58.2	<0.001	76.5	45.7	0.363	25.1	41.6	0.472	42.8	41.1	0.020
Social security	37.5	38.3	61.7		N/A			65.1	41.2		47.4	44.4	
Private	20.8	48.5	51.5		23.5	49.6		9.8	32.0		9.8	60.4	
**Chronic disease**													
Yes	38.0	29.2	70.8	<0.001	33.6	22.5	<0.001	32.9	27.0	<0.001	31.5	34.6	0.001
No	62.0	49.6	50.4		66.4	58.8		67.1	47.0		68.5	49.2	
**Excellent or very good SRH**		41.9	58.1			46.6			40.4			44.6	

Weighted percent values are double-weighted by stabilized inverse probability weights and survey weights. *p*-Values are for chi-squared test between SRH and each covariate.

SRH, self-rated health.

**Table 2:** Characteristics of the population according to SRH status: Jamaica, Mexico, and Panama.

Characteristic	Jamaica			Mexico			Panama		
	Total	Excellent or very good SRH	*p*-Value	Total	Excellent or very good SRH	*p*-Value	Total	Excellent or very good SRH	*p*-Value
**Number of observations**	1,140	602		1,182	414		1,083	451	
**Weighted population**	1,142	598		1,173	346		1,058	421	
**Variables (weighted percent)**									
**Sex**									
Female	53.3	46.5	<0.001	51.8	26.4	0.083	51.6	37.1	0.147
Male	46.7	59.1		48.2	32.9		48.4	42.6	
**Age (years)**									
20–25	22.1	51.1	0.146	23.8	47.2	<0.001	19.2	60.9	<0.001
26–45	42.4	55.1		47.1	28.1		39.8	42.0	
46–59	19.4	55.2		21.8	21.4		20.8	33.5	
≥60	16.1	43.8		7.3	5.4		20.2	21.8	
**Schooling**									
Elementary school or less	25.9	48.7	0.499	26.4	22.3	0.017	37.5	26.8	<0.001
Secondary school	48.2	52.8		53.1	29.3		27.2	47.9	
College	19.3	54.3		18.2	36.6		22.5	49.6	
Not specified	6.6	58.1		2.3	60.5		12.8	43.4	
**Health insurance**									
Government	61.5	48.5	0.004	33.1	26.7	0.389	16.7	35.8	0.431
Social security	N/A			48.9	29.7		62.4	39.4	
Private	38.5	58.6		18.0	34.1		20.9	44.1	
**Chronic disease**									
Yes	52.2	42.0	<0.001	35.5	15.7	<0.001	38.4	27.8	<0.001
No	47.8	63.8		64.5	37.1		61.6	47.3	
**Excellent or very good SRH**		52.4			29.5			39.8	

Weighted percent values are double-weighted by stabilized inverse probability weights and survey weights. *p*-Values are for chi-squared test between SRH and each covariate.

N/A, no applicable; SRH, self-rated health.

Tables 3 and 4 show the participants’ experience with PC services in the full sample and by country. The proportion of participants who reported that the PC facility was easy to contact by telephone during regular office hours ranged from 38.1% in El Salvador to 75.2% in Jamaica. Patients from Brazil reported less frequently that the PC provider spent enough time with them (31.8%), while in Colombia this figure reached 74.2%. Regarding patient–provider communication, the opportunity to ask questions and having the PC provider explain things in a way that was easy to understand were less frequent in Brazil (58.0% and 63.9%, respectively) and more frequent in Mexico (79.5% had the opportunity to ask questions) and Colombia (81.3% received explanations in a way that was easy to understand). Relating to the technical quality of care, only 40.9% in Brazil reported that the PC provider knew relevant information about their medical history, while this figure was 75.4% in Mexico. Only between 25.9% (in Jamaica) and 44.2% (in Panama) reported that the PC provider talked about healthy lifestyles, while between 25.8% and 26% (in Panama and El Salvador) and 40.7% and 40.2% (in Brazil and Mexico) had their preventive exams up to date. The percentage of participants who considered that the PC provider solved most of their health problems ranged from 54.2% in Brazil to 80.6% in Mexico, while only from 21.8% (in Brazil) to 45.4% (in Mexico) stated that the PC provider helped to coordinate healthcare. The average OPCE score ranged from 0.44 points in Brazil to 0.63 points in Mexico.

**Table 3:** Patients’ experience with PC according to SRH status: 6-country sample, Brazil, Colombia, and El Salvador.

Domain and variable (weighted percent)	6-country sample				Brazil			Colombia			El Salvador		
	Total	Excellent or very good SRH	Good, fair, or poor SRH	*p*-Value	Total	Excellent or very good SRH	*p*-Value	Total	Excellent or very good SRH	*p*-Value	Total	Excellent or very good SRH	*p*-Value
**Number of observations**	6,100	2,651	3,449		874	413		1,009	407		812	364	
**Weighted population**	6,007	2,516	3,491		857	399		1,000	404		777	346	
**I. Contact with PC clinic**													
PC facility is easy to contact by telephone during regular office hours													
Yes	50.9	46.9	53.1	<0.001	50.3	46.6	0.945	44.1	46.5	0.010	38.1	52.9	0.006
No	42.0	36.7	63.3		42.6	47.0		40.2	32.0		57.0	39.0	
Never tried to contact PC facility	7.1	36.5	63.5		7.1	44.1		15.7	45.0		4.9	44.3	
**II. Time spent with provider**													
PC provider spends enough time with patient													
Yes	59.6	43.8	56.2	0.005	31.8	49.5	0.331	74.2	43.3	0.023	60.4	46.8	0.178
No	40.4	39.1	60.9		68.2	45.3		25.8	32.2		39.6	41.1	
**III. Patient–provider communication**													
PC provider gives an opportunity to ask questions													
Yes	71.4	43.8	56.2	<0.001	58.0	53.8	<0.001	77.5	43.9	0.004	70.2	45.4	0.526
No	28.6	37.0	63.0		42.0	36.7		22.5	28.3		29.8	42.6	
PC provider explains things in a way that is easy to understand													
Yes	73.3	44.1	55.9	<0.001	63.9	51.2	0.005	81.3	43.1	0.008	71.2	45.7	0.424
No	26.7	35.9	64.1		36.1	38.6		18.7	28.7		28.8	41.9	
**IV. Technical quality and solving problems**													
PC provider always or often knows relevant information about the patient’s medical history													
Yes	66.0	43.1	56.9	0.042	40.9	42.5	0.105	74.4	43.4	0.032	67.3	46.2	0.260
No	34.0	39.5	60.5		59.1	49.4		25.6	31.6		32.7	41.2	
PC provider advises about healthy lifestyles (healthy food, regular physical activity, and possible stressors)													
Yes	35.5	41.4	58.6	0.631	28.6	46.5	0.977	28.5	43.9	0.322	42.1	47.2	0.264
No	64.5	42.2	57.8		71.4	46.7		71.5	39.0		57.9	42.7	
Preventive exams up to date													
Yes	33.0	40.5	59.5	0.239	40.7	42.5	0.103	31.4	43.9	0.294	26.0	40.7	0.259
No	67.0	42.6	57.4		59.3	49.4		68.6	38.8		74.0	45.9	
PC provider solves most of the patient’s health problems													
Yes	69.6	44.3	55.7	<0.001	54.2	51.1	0.021	74.8	45.3	<0.001	71.7	46.6	0.136
No	30.4	36.5	63.5		45.8	41.3		25.2	25.9		28.3	39.6	
**V. Healthcare coordination**													
PC provider always or often helps to coordinate care with other physicians or sources of care													
Yes	36.3	46.1	53.9	0.001	21.8	56.0	0.692	40.4	46.2	0.030	40.9	52.5	0.007
No	57.6	39.6	60.4		77.9	47.3		46.0	39.2		55.6	39.3	
Not necessary to coordinate care	6.1	38.3	61.7		0.3	57.8		13.6	27.3		3.5	35.7	
**OPCE score, mean (95% CI)**	0.56 (0.55–0.57)	0.58 (0.57–0.60)	0.54 (0.53–0.55)	<0.001	0.44 (0.41–0.46)	0.45 (0.42–0.48)	0.177	0.60 (0.58–0.62)	0.65 (0.62–0.68)	<0.001	0.55 (0.52–0.57)	0.58 (0.55–0.61)	0.014

Weighted percent values are double-weighted by stabilized inverse probability weights and survey weights. *p*-Values are for chi-squared test between SRH and each independent variable, or Student *t* test for comparison of OPCE score between people who reported excellent or very good SRH and those who reported good, fair, or poor (or not sure) SRH.

OPCE, overall patient-centered primary care experience; PC, primary care; SRH, self-rated health.

**Table 4:** Patients’ experience with PC according to SRH status: Jamaica, Mexico, and Panama.

Domain and variable (weighted percent)	Jamaica			Mexico			Panama		
	Total	Excellent or very good SRH	*p*-Value	Total	Excellent or very good SRH	*p*-Value	Total	Excellent or very good SRH	*p*-Value
**Number of observations**	1,140	602		1,182	414		1,083	451	
**Weighted population**	1,142	598		1,173	346		1,058	421	
**I. Contact with PC clinic**									
PC facility is easy to contact by telephone during regular office hours									
Yes	75.2	54.1	0.218	45.8	37.3	<0.001	46.4	42.0	0.284
No	23.8	46.8		45.6	23.9		48.2	38.9	
Never tried to contact PC facility	1.0	61.3		8.6	18.0		5.4	28.4	
**II. Time spent with provider**									
PC provider spends enough time with patient									
Yes	58.0	55.1	0.070	73.8	33.5	<0.001	53.8	41.6	0.323
No	42.0	48.7		26.2	18.3		46.2	37.7	
**III. Patient–provider communication**									
PC provider gives an opportunity to ask questions									
Yes	63.9	56.0	0.007	79.5	32.0	0.019	76.6	39.2	0.548
No	36.1	45.9		20.5	20.0		23.4	41.8	
PC provider explains things in a way that is easy to understand									
Yes	67.6	56.8	<0.001	78.3	33.8	<0.001	75.7	38.6	0.273
No	32.4	43.2		21.7	14.2		24.3	43.6	
**IV. Technical quality and solving problems**									
PC provider always or often knows relevant information about the patient’s medical history									
Yes	58.6	55.7	0.025	75.4	33.7	<0.001	75.3	40.8	0.349
No	41.4	47.7		24.6	16.9		24.7	36.7	
PC provider advises about healthy lifestyles (healthy food, regular physical activity, and possible stressors)									
Yes	25.9	48.2	0.157	43.6	30.3	0.696	44.2	40.7	0.680
No	74.1	53.9		56.4	28.9		55.8	39.1	
Preventive exams up to date									
Yes	33.0	55.8	0.186	40.2	25.8	0.108	25.8	38.3	0.629
No	67.0	50.7		59.8	32.0		74.2	40.3	
PC provider solves most of the patient’s health problems									
Yes	59.0	58.4	<0.001	80.6	32.7	<0.001	74.6	39.3	0.671
No	41.0	43.8		19.4	16.5		25.4	41.2	
**V. Healthcare coordination**									
PC provider always or often helps to coordinate care with other physicians or sources of care									
Yes	31.4	56.9	0.025	45.4	37.6	<0.001	36.1	43.5	0.282
No	63.6	49.2		46.7	19.7		59.0	38.1	
Not necessary to coordinate care	5.0	64.8		7.9	41.2		4.9	33.7	
**OPCE score, mean (95% CI)**	0.53 (0.51–0.55)	0.56 (0.54–0.59)	<0.001	0.63 (0.61–0.65)	0.71 (0.68–0.74)	<0.001	0.57 (0.55–0.59)	0.58 (0.55–0.61)	0.603

Weighted percent values are double-weighted by stabilized inverse probability weights and survey weights. *p*-Values are for chi-squared test between SRH and each independent variable, or Student *t* test for comparison of OPCE score between people who reported excellent or very good SRH and those who reported good, fair, or poor (or not sure) SRH.

OPCE, overall patient-centered primary care experience; PC, primary care; SRH, self-rated health.

In bivariable analyses, the average OPCE score was significantly higher in participants with excellent or very good SRH in 4 out of 6 countries. For specific features of patient-centered PC, the proportion of respondents with excellent or very good SRH was significantly higher among those who had a PC facility that was easy to contact in Colombia, El Salvador, and Mexico; who reported that the PC provider spent enough time with them in Colombia and Mexico; who had the opportunity to ask questions in Brazil, Colombia, and Mexico; who had a PC provider who explained things in a way that was easy to understand in Brazil, Colombia, Jamaica, and Mexico; who perceived that the PC provider knew relevant information about their medical history in Colombia, Jamaica, and Mexico; who considered that PC provider solved most of their health problems in Brazil, Colombia, Jamaica, and Mexico; and who reported that PC provider coordinated care with other providers or sources of care in Colombia, El Salvador, Jamaica, and Mexico.

Table 5 shows the results of the pooled multiple Poisson regression model double-weighted by survey and stabilized IP weights to test the association of OPCE score with excellent or very good SRH. The coefficients represent prevalence ratios of the report of excellent or very good SRH; their interpretation is the same as for risk ratios. Assessment of interaction between countries and OPCE score identified a significant positive interaction in Mexico (RERI 0.55, 95% CI 0.09–1.02, *p =* 0.019) (S2 Table); we included an interaction term in the analytic model (Table 5). After adjustment for socio-demographic and health covariates, in all countries except Mexico, patients with an OPCE score of 1 were 1.6 times (95% CI 1.37–1.90, *p <* 0.001) as likely to report excellent or very good SRH as those with a score of 0. The association was significantly stronger in Mexico: incorporating the interaction term, patients with an OPCE score of 1 had a 4.27 (95% CI 2.34–7.81, *p <* 0.001) times higher probability of reporting excellent or very good SRH compared to those with an OPCE score of 0.

**Table 5:** Association of OPCE score with excellent and very good self-rated health (*n* = 6,100).

Variable	aPR (95% CI)	*p*-Value
**OPCE score**	**1.61 (1.37–1.90)**	<0.001
**Interaction term between Mexico and OPCE score**	**2.65 (1.42–4.95)**	0.002
**Covariates**		
**Female**	**0.88 (0.82–0.95)**	0.001
**Age (years)**		
26–45	**0.85 (0.78–0.93)**	<0.001
46–59	**0.80 (0.72–0.90)**	<0.001
≥60	**0.64 (0.54–0.75)**	<0.001
**Education**		
Elementary school or less	Ref	
Secondary school	0.99 (0.89–1.11)	0.924
College	1.12 (0.99–1.25)	0.053
Not specified	1.02 (0.86–1.20)	0.839
**Health insurance**		
Government	Ref	
Social security	1.04 (0.92–1.17)	0.509
Private	1.05 (0.95–1.15)	0.316
**Chronic disease**	**0.61 (0.56–0.68)**	<0.001
**Country**		
Brazil	**1.25 (1.08–1.44)**	0.003
Colombia	0.96 (0.83–1.11)	0.621
El Salvador	1.08 (0.95–1.23)	0.216
Jamaica	**1.45 (1.27–1.65)**	<0.001
Mexico	**0.36 (0.22–0.58)**	<0.001
Panama	Ref	

All prevalence ratios were adjusted by the covariates presented in this table. Bold values highlight the statistically significant aPRs.

aPR, adjusted prevalence ratio; OPCE, overall patient-centered primary care experience; PC, primary care; SRH, self-rated health.

Tables 6 and 7 depict the association of specific PC patient experiences with excellent or very good SRH. We found evidence of multiple interactions between countries and specific features of patient-centered PC (S2 Table); we thus present results stratified by country (Tables 6 and 7). The analysis revealed differences among countries in patient experiences associated with a high probability of having excellent or very good SRH, when controlling for the study covariates. After adjustment for socio-demographic and health characteristics, the experience of easy contact with the PC facility by telephone during regular office hours was associated with excellent or very good SRH in Mexico (aPR 1.35, 95% CI 1.04–1.74, *p =* 0.023), the perception that the PC provider gives an opportunity to ask questions was associated with excellent or very good SRH in Brazil (aPR 1.42, 95% CI 1.11–1.83, *p =* 0.006), having a PC provider who knows relevant information about the patient’s medical history was associated with excellent or very good SRH in Mexico (aPR 1.47, 95% CI 1.03–2.12, *p =* 0.036) but was negatively associated with excellent or very good SRH in Brazil (aPR 0.71, 95% CI 0.56–0.89, *p =* 0.003), the perception that the PC provider solves most of the patient’s health problems was associated with excellent or very good SRH in Colombia (aPR 1.38, 95% CI 1.01–1.91, *p =* 0.046) and in Jamaica (aPR 1.21, 95% CI 1.02–1.43, *p =* 0.030), and coordination of care by the PC provider was associated with excellent or very good SRH in Mexico (aPR 1.53, 95% CI 1.19–1.98, *p =* 0.001). After adjustment for covariates, no individual features of patient-centered PC were associated with excellent or very good SRH in El Salvador or Panama.

**Table 6:** Association of specific PC patient experiences with excellent or very good self-rated health: Brazil, Colombia, and El Salvador.

Domain and variable	Brazil *n =* 874		Colombia *n =* 1,009		El Salvador *n =* 812	
	aPR (95% CI)	*p*-Value	aPR (95% CI)	*p*-Value	aPR (95% CI)	*p*-Value
**I. Contact with clinic**						
PC facility is easy to contact by telephone during regular office hours						
Yes	1.04 (0.85–1.27)	0.680	1.25 (0.98–1.61)	0.072	1.18 (0.98–1.42)	0.089
No	Ref		Ref		Ref	
Never tried to contact PC facility	0.90 (0.67–1.20)	0.472	**1.39 (1.00–1.92)**	0.049	1.10 (0.73–1.67)	0.647
**II. Time spent with provider**						
PC provider spends enough time with patient	1.08 (0.89–1.31)	0.448	1.01 (0.76–1.34)	0.969	1.01 (0.78–1.29)	0.945
**III. Patient–provider communication**						
PC provider gives an opportunity to ask questions	**1.42 (1.11–1.83)**	0.006	1.10 (0.77–1.57)	0.591	0.93 (0.71–1.22)	0.607
PC provider explains things in a way that is easy to understand	1.09 (0.85–1.39)	0.497	1.08 (0.78–1.49)	0.641	0.97 (0.71–1.34)	0.878
**IV. Technical quality and solving problems**						
PC provider knows relevant information about the patient’s medical history	**0.71 (0.56–0.89)**	0.003	1.08 (0.79–1.47)	0.649	0.78 (0.79–1.34)	0.887
PC provider advises about healthy lifestyles (nutrition, physical activity, stressors)	1.10 (0.93–1.30)	0.253	1.12 (0.89–1.40)	0.322	1.11 (0.93–1.34)	0.240
Preventive exams up to date	1.02 (0.83–1.25)	0.852	0.97 (0.76–1.23)	0.790	0.83 (0.67–1.05)	0.118
PC provider solves most of the patient’s health problems	1.14 (0.93–1.38)	0.205	**1.38 (1.01–1.91)**	0.046	1.16 (0.87–1.55)	0.295
**V. Care coordination**						
PC provider helps to coordinate care with other physicians or sources of care						
Yes	0.89 (0.70–1.13)	0.330	0.98 (0.78–1.23)	0.855	1.17 (0.98–1.41)	0.088
No	Ref		Ref		Ref	
It was not necessary to coordinate care	1.18 (0.55–2.52)	0.673	0.66 (0.42–1.02)	0.064	0.90 (0.49–1.64)	0.734
**Covariates**						
**Female**	1.01 (0.85–1.22)	0.849	**0.76 (0.61–0.95)**	0.018	0.91 (0.76–1.10)	0.345
**Age (years)**						
18–25	Ref		Ref		Ref	
26–45	0.98 (0.80–1.19)	0.839	1.05 (0.82–1.36)	0.685	**0.79 (0.64–0.98)**	0.030
46–59	**0.60 (0.42–0.85)**	0.004	1.20 (0.88–1.64)	0.248	0.99 (0.77–1.28)	0.968
≥60	**0.39 (0.23–0.66)**	0.001	1.02 (0.62–1.69)	0.926	0.92 (0.68–1.23)	0.570
**Education**						
Elementary school or less	Ref		Ref		Ref	
Secondary school	0.90 (0.75–1.07)	0.243	1.08 (0.76–1.52)	0.679	0.97 (0.76–1.24)	0.835
College	1.04 (0.79–1.39)	0.763	1.24 (0.86–1.78)	0.251	1.11 (0.89–1.38)	0.370
Not specified	1.23 (0.63–2.41)	0.545	0.56 (0.16–2.02)	0.378	0.79 (0.59–1.07)	0.130
**Health insurance**						
Government	Ref		Ref		Ref	
Social security	N/A		0.98 (0.75–1.29)	0.910	1.03 (0.84–1.26)	0.767
Private	1.14 (0.91–1.44)	0.251	0.71 (0.46–1.08)	0.110	**1.31 (1.03–1.68)**	0.029
**Chronic disease diagnosis**	**0.53 (0.40–0.71)**	<0.001	**0.65 (0.48–0.87)**	0.004	**0.72 (0.58–0.90)**	0.004

Multiple Poisson regression models double-weighted by survey and stabilized inverse variance weights and stratified by country. All prevalence ratios were adjusted by the covariates presented in this table. Bold values highlight the statistically significant aPRs.

aPR, adjusted prevalence ratio; PC, primary care.

**Table 7:** Association of specific PC patient experiences with excellent or very good self-rated health: Jamaica, Mexico, and Panama.

Domain and variable	Jamaica *n =* 1,140		Mexico *n =* 1,182		Panama *n =* 1,083	
	aPR (95% CI)	*p*-Value	aPR (95% CI)	*p*-Value	aPR (95% CI)	*p*-Value
**I. Contact with clinic**						
PC facility is easy to contact by telephone during regular office hours						
Yes	0.99 (0.83–1.19)	0.938	**1.35 (1.04–1.74)**	0.023	1.06 (0.88–1.27)	0.550
No	Ref		Ref		Ref	
Never tried to contact PC facility	1.05 (0.57–1.94)	0.862	0.89 (0.51–1.53)	0.663	0.79 (0.46–1.36)	0.397
**II. Time spent with provider**						
PC provider spends enough time with patient	0.94 (0.80–1.11)	0.504	1.14 (0.83–1.57)	0.437	1.00 (0.82–1.22)	1.000
**III. Patient–provider communication**						
PC provider gives an opportunity to ask questions	1.10 (0.93–1.30)	0.249	0.76 (0.48–1.16)	0.199	0.91 (0.72–1.14)	0.405
PC provider explains things in a way that is easy to understand	1.16 (0.96–1.41)	0.124	1.35 (0.82–2.20)	0.235	0.82 (0.66–1.02)	0.074
**IV. Technical quality and solving problems**						
PC provider knows relevant information about the patient’s medical history	0.98 (0.83–1.15)	0.764	**1.47 (1.03–2.12)**	0.036	1.24 (0.97–1.58)	0.085
PC provider advises about healthy lifestyles (nutrition, physical activity, stressors)	0.93 (0.80–1.08)	0.343	1.12 (0.88–1.44)	0.351	1.02 (0.85–1.21)	0.852
Preventive exams up to date	0.96 (0.82–1.13)	0.655	0.93 (0.71–1.22)	0.629	1.08 (0.87–1.35)	0.489
PC provider solves most of the patient’s health problems	**1.21 (1.02–1.43)**	0.030	1.21 (0.75–1.94)	0.428	1.00 (0.78–1.26)	0.976
**V. Care coordination**						
PC provider helps to coordinate care with other physicians or sources of care						
Yes	1.06 (0.92–1.23)	0.424	**1.53 (1.19–1.98)**	0.001	1.17 (0.96–1.42)	0.118
No	Ref		Ref		Ref	
It was not necessary to coordinate care	**1.24 (1.00–1.54)**	0.049	**1.91 (1.34–2.73)**	<0.001	1.02 (0.65–1.59)	0.926
**Covariates**						
**Female**	**0.81 (0.69–0.94)**	0.005	**0.78 (0.61–0.98)**	0.037	0.94 (0.78–1.14)	0.551
**Age (years)**						
18–25	Ref		Ref		Ref	
26–45	1.08 (0.92–1.26)	0.374	**0.65 (0.51–0.83)**	0.001	**0.72 (0.60–0.88)**	0.001
46–59	1.19 (0.97–1.46)	0.100	**0.53 (0.36–0.78)**	0.001	**0.65 (0.50–0.85)**	0.002
≥60	0.95 (0.74–1.22)	0.682	**0.19 (0.04–0.80)**	0.024	**0.48 (0.32–0.74)**	0.001
**Education**						
Elementary school or less	Ref		Ref		Ref	
Secondary school	0.95 (0.79–1.15)	0.619	0.92 (0.66–1.28)	0.615	**1.41 (1.06–1.89)**	0.019
College	0.97 (0.80–1.17)	0.758	1.22 (0.84–1.78)	0.299	**1.49 (1.12–1.98)**	0.006
Not specified	1.07 (0.82–1.39)	0.611	1.57 (0.81–3.05)	0.181	**1.38 (1.01–1.90)**	0.044
**Health insurance**						
Government	Ref		Ref		Ref	
Social security	N/A		1.13 (0.85–1.50)	0.390	1.02 (0.76–1.39)	0.876
Private	1.06 (0.97–1.26)	0.120	1.15 (0.82–1.60)	0.416	1.03 (0.74–1.44)	0.853
**Chronic disease diagnosis**	**0.68 (0.59–0.78)**	<0.001	**0.54 (0.38–0.76)**	<0.001	**0.75 (0.59–0.97)**	0.026

Multiple Poisson regression models double-weighted by survey and stabilized inverse variance weights and stratified by country. All prevalence ratios were adjusted by the covariates presented in this table. Bold values highlight the statistically significant aPRs.

aPR, adjusted prevalence ratio; PC, primary care.

## Discussion

This secondary analysis of a nationally representative survey of the urban population in 6 LAC countries found that higher OPCE was associated with excellent or very good SRH. At the same time, the specific features of patient-centered PC associated with excellent or very good SRH differed among countries, with features from the domains of contact with clinic, communication, technical quality, and coordination showing significant associations in at least 1 country. The findings underscore the importance of high-quality, patient-centered PC as a path to improved population health while identifying areas for future country-specific investigation.

Overall scores are considered a valid alternative to global ratings in patient experience surveys [27]. To our knowledge there were at least 2 previous studies in the US and South Korea that investigated the association between overall PC quality metrics and SRH [20,21]. The first study utilized summary metrics of accessibility, interpersonal relationships, and continuity, while the second included first contact, personalized care, coordination function, comprehensiveness, and family/community orientation. Both measures showed a positive association between better PC experience and better SRH. Consistent with these 2 studies, we found a significant association of the average OPCE score with excellent or very good SRH in the context of LMICs in the LAC region.

Interestingly, we found that in Mexico patients with an OPCE score of 1 had a 4.27 times higher probability of reporting excellent or very good SRH compared to those with an OPCE score of 0. Also, we found that the overall SRH in Mexico was substantially lower than in the other countries, and the factors that explain this difference might also help us understand why the relationship of SRH with patient-centered PC is stronger in Mexico. Further country-specific research would be needed to identify such factors.

While broad policy statements on the centrality of PC for achieving health for all are important [2], these often lack the specific guidance to policy-makers who intend to pursue health system reform and introduce PC orientation within their health systems. Thus, detailed knowledge on specific patient experiences associated with better SRH (i.e., excellent or very good SRH) is important to identify priority areas for improvement in the delivery of healthcare, together with further assessments with longitudinal or experimental data [43]. In our study, PC features associated with excellent or very good SRH varied among countries. Two countries (El Salvador and Panama) showed no significant associations for the individual features of patient-centered PC, suggesting that the totality of the experience was more salient than any component within it. In Mexico, having a facility easy to contact by telephone, having a provider who knows relevant information about the patient’s medical history, and having a provider who coordinates healthcare were associated with better SRH. Having a PC provider who gives an opportunity to ask questions was associated with better SRH in Brazil, and having a PC provider who solves most health problems was associated with better SRH in Colombia and in Jamaica. Taken as a whole, the results suggest that the domains of patient-centered PC are all important to patient-reported health, but that the individual components with greatest relevance vary across settings. These characteristics shape the definition, goals, and priorities of PC. The attainment of PC goals requires easy communication with the clinic or provider to guarantee timely access to care, coordination among healthcare providers to assure continuity of care, and the ability to solve health-related problems. Previous studies found the importance of these experiences to patients [44,45], yet, to the best of our knowledge, our study is the first to find the association of these characteristics with very good or excellent SRH. Effective patient-centered communication was associated with improved health outcomes in several studies [46,47]. In our study, the opportunity to ask questions was significant only in Brazil.

The study has several limitations. First, it is an observational analysis of a cross-sectional survey, which does not allow making causal inferences or identifying the direction of the association between the study variables. Bidirectional relationships could be possible between better SRH and some healthcare experiences. For instance, on the one hand, people with poorer health are less likely to give the clinician credit for solving issues, and on the other hand, worse health problems are harder to solve. Second, due to the high prevalence of missing data, the analysis included IP weighting; therefore, we had to assume that the population with a regular PC clinic or doctor was exchangeable, conditional on covariates, with the population without a regular PC clinic or doctor; if this assumption was violated, the results would not be generalizable to those without a regular PC clinic or doctor. Third, in cross-national comparisons of survey data, cultural differences may lead to different interpretations of the questions being asked of respondents. For this reason, questionnaires had to be adapted for the characteristics of each country. Rather than focusing on the specifics of service provision in each country, this study aimed at identifying the broader roles of PC that may affect patient experience. Fourth, the results of our study are generalizable only to the urban populations of the analyzed 6 countries, as the samples were designed to represent national urban populations in each country. The results do not represent experiences of rural populations. Fifth, our findings cannot be generalized to other LMICs because of the different characteristics of their healthcare systems. Finally, information on type of employment and income was not collected by the survey; however, information on level of education and HI was available and included in this study.

### Conclusion

In the context of the 40th anniversary of the Declaration of Alma-Ata, there seems to be broad consensus that strengthening PC is an essential strategy to achieve universal health coverage and the Sustainable Development Goals. In parallel, there is a growing interest in the importance of patient-centered healthcare as a tool for improving outcomes. However, to date there is little empirical cross-country evidence from LMICs that tests whether the main attributes of patient-centered PC are associated with better individual health. This study contributes to closing this gap by showing specific characteristics of patient-centered PC, and an overall summary measure of patient-centered PC performance, that are associated with better SRH in a sample representative of nearly 330 million people in 6 LAC countries. While the current study focused on self-reported cross-sectional data, the expansion of PC coverage in LAC countries and the increasing availability of administrative and clinical data associated with the introduction of electronic health records should allow for more longitudinal analyses to be conducted in the future.

## Supporting information

S1 STROBE Checklist (DOC)

S1 Table Missing data by country. (DOCX)

S2 Table Results of the tests for additive interactions: Relative excess risk due to interaction and 95% CI. (DOCX)

## Abbreviations

aPR: adjusted prevalence ratio
HI: health insurance
IP: inverse probability
LAC: Latin American and Caribbean
LMICs: low and middle-income countries
OPCE: overall patient-centered primary care experience
PC: primary care
RERI: relative excess risk due to interaction
SRH: self-rated health
