# Supplementary material for: Patient-centered primary care and self-rated health in 6 Latin American and Caribbean countries: Analysis of a public opinion cross-sectional survey
Source: PLoS Med. 2018 Oct 9;15(10):e1002673. doi: 10.1371/journal.pmed.1002673 (PMC6177127; doi:10.1371/journal.pmed.1002673)
Supplement: S1 STROBE Checklist — (DOC) [file pmed.1002673.s001.doc]

STROBE Statement—Checklist of items that should be included in reports of ***cross-sectional studies***

**Article: ”****Patient-centered primary care and self-rated health in six Latin American and Caribbean countries: analysis of a public opinion cross-sectional survey”**

|  | Item No | | Recommendation | Text excerpted from the manuscript and reported on section or paragraph # |  |
| --- | --- | --- | --- | --- | --- |
| **Title and abstract** | 1 | (*a*) Indicate the study’s design with a commonly used term in the title or the abstract | | The title and the abstract specify study’s design. Title: ”Patient-centered primary care and self-rated health in six Latin American and Caribbean countries: analysis of a public opinion cross-sectional survey” Abstract (Methods and findings section): We conducted a secondary analysis of a 2013 public opinion cross-sectional survey on perceptions and experiences with healthcare systems in Brazil, Colombia, El Salvador, Jamaica, Mexico, and Panama | |
|  |  | (*b*) Provide in the abstract an informative and balanced summary of what was done and what was found | | The abstract provides an informative and balanced summary of what was done and what was found. | |
| Introduction | | | |  | |
| Background/rationale | 2 | Explain the scientific background and rationale for the investigation being reported | | The introduction section explains the scientific background and rationale for the investigation being reported (introduction, paragraphs 1-3) | |
| Objectives | 3 | State specific objectives, including any prespecified hypotheses | | The objectives of the present study were: 1) to test the association between overall patient-centered PC experience and SRH in six Latin American and Caribbean countries (LAC) and 2) to identify specific features of patient-centered PC associated with better SRH. (introduction section, last paragraph) | |
| Methods | | | |  | |
| Study design | 4 | Present key elements of study design early in the paper | | We presented key elements of study design early in the paper (methods section, paragraph 1) | |
| Setting | 5 | Describe the setting, locations, and relevant dates, including periods of recruitment, exposure, follow-up, and data collection | | We specified that the survey was conducted in Brazil, Colombia, El Salvador, Jamaica, Mexico, and Panama and included a nationally representative urban sample of the population that comprised between 1500 and 1506 adults per country. During 2012 and 2013, Harris Interactive collected the data through telephone interviews. The survey used an adapted version of the methodology and questionnaire that the Commonwealth Fund has been applying in Europe, Australia, Canada and the United States over the past 15 years. (methods section, paragraphs 1-2) | |
| Participants | 6 | (*a*) Give the eligibility criteria, and the sources and methods of selection of participants | | We specified that the selection criteria considered any household member 18+ years. Only one adult per household was interviewed. (methods section, paragraph 2) | |
| Variables | 7 | Clearly define all outcomes, exposures, predictors, potential confounders, and effect modifiers. Give diagnostic criteria, if applicable | | We specified that the study dependent variable was “excellent and very good self-rated health”, obtained from the general self-rated health report categorized as one = “excellent and very good” and zero= “good, fair, poor and not sure.” (methods section, study variables, paragraph 1) We selected items related to primary care that fall into the domains of patient-centered healthcare identified in the literature [10,23-25] and organized them by domain: I. Contact with PC clinic (PC facility is easy to contact by telephone during regular office hours).II. Time spent (PC provider spends enough time with patient)III. Patient-provider communication (PC provider gives an opportunity to ask questions; PC provider explains things in a way that is easy to understand)IV. Technical quality and solving problems (PC provider always or often knows relevant information about the patient’s medical history; PC provider advices about healthy lifestyles (health food, regular physical activity, and possible stressors); Preventive exams up to date; PC provider solves most of health problems)V. Healthcare coordination (PC physician/staff at the source of care always or often helps to coordinate care with other physicians or sources of care).All PC variables were measured on a five-options Likert scale and categorized as one = Yes (always or often) and zero=No (sometimes, rarely or not and not sure).We calculated overall patient-centered PC experience (OPCE) as the arithmetic mean of these items, following the recommendation of previous research on the use of patient experience surveys to assess service provision. OPCE score ranges from zero to one, where zero means that participant did not have any of the nine patient-centered PC experiences, while one means that he/she reported having all these experiences. Furthermore, based on survey data availability, we included the following covariates: sex, age, schooling, chronic disease, and the type of health insurance. (methods section, study variables, paragraphs 2-5) | |
| Data sources/ measurement | 8* | For each variable of interest, give sources of data and details of methods of assessment (measurement). Describe comparability of assessment methods if there is more than one group | | We specified that the study used data from 2013 public opinion cross-sectional survey on perceptions and experiences with healthcare systems in Brazil, Colombia, El Salvador, Jamaica, Mexico, and Panama. (methods section, paragraph 1) | |
| Bias | 9 | Describe any efforts to address potential sources of bias | | We applied a double-weighted strategy with the use of stabilized inverse probability (IP) weights and survey weights to adjust the analysis for sample weights and to correct for potential selection bias (methods section, statistical analysis, paragraph 2) | |
| Study size | 10 | Explain how the study size was arrived at | | In our secondary data analysis, we used existing survey sample that comprised between 1500 and 1506 adults per country. (methods section, paragraph 1) Also, S1 Table presents the information on the original sample, missing data and the analytical sample. | |
| Quantitative variables | 11 | Explain how quantitative variables were handled in the analyses. If applicable, describe which groupings were chosen and why | | We specified that the study dependent variable was “excellent and very good self-rated health”, obtained from the general self-rated health report categorized as one = “excellent and very good” and zero= “good, fair, poor and not sure.” We specified that all PC variables were measured on a five-options Likert scale and categorized as one = Yes (always or often) and zero=No (sometimes, rarely or not and not sure). Furthermore, to achieve the second objective of the study, we calculated overall patient-centered PC experience (OPCE) as the arithmetic mean of these items, following the recommendation of previous research on the use of patient experience surveys to assess service provision. OPCE score ranges from zero to one, where zero means that participant did not have any of the nine patient-centered PC experiences, while one means that he/she reported having all these experiences. We assumed that each component of OPCE score contributes equally to patients’ experiences and a difference in patient experiences has a constant effect on SRH. (methods section, study variables, paragraphs 1-5) | |
| Statistical methods | 12 | (*a*) Describe all statistical methods, including those used to control for confounding | | We used descriptive statistics to analyze the characteristics and PC experiences of the study participants. We performed a bivariate analysis including chi-square test between dependent variable (SRH) and each independent variable (PC experiences), or categorical covariate. We used Student t-tests for comparison of continuous variables (OPCE score) between people with excellent and very good SRH and those who reported good, fair and poor SRH.  We used multiple Poisson regression model double-weighted by survey and stabilized IP weights 1) to test the association between overall patient-centered PC experience and SRH in six Latin American and Caribbean countries (LAC) and 2) to identify specific features of patient-centered PC associated with better SRH. (methods section, statistical analysis, paragraphs 1-3) | |
|  |  | (*b*) Describe any methods used to examine subgroups and interactions | | To examine subgroups we performed a bivariate analysis including chi-square test between dependent variable (SRH) and each independent variable (PC experiences), or categorical covariate. We used Student t-tests for comparison of continuous variables (OPCE score) between people with excellent and very good SRH and those who reported good, fair and poor SRH.  We initially fit pooled models across all six countries, and then calculated the relative excess risk due to interaction (RERI) as a measure of interaction on the additive scale; additive interaction is more indicative of underlying causal interaction than interaction on the relative (ratio) scale. Where evidence of interaction was identified (RERI significant at p≤0.05), we include interaction terms for country or stratified the model in the case of multiple interactions identified. (methods section, statistical analysis, paragraph 3) | |
|  |  | (*c*) Explain how missing data were addressed | | To address potential bias due to the missing data in the statistical analysis we applied a double-weighted strategy with the use of stabilized inverse probability (IP) weights. (methods section, statistical analysis, paragraph 2) | |
|  |  | (*d*) If applicable, describe analytical methods taking account of sampling strategy | | The double-weighted strategy that we applied included survey weights in order to account for survey sampling strategy. (methods section, statistical analysis, paragraph 2) | |
|  |  | (*e*) Describe any sensitivity analyses | | We performed a sensitivity analysis in which the IP weights were calculated after the individuals without regular primary care were dropped. The results were similar to the main analysis, suggesting that our findings were not distorted by including everyone when calculating the IP weights. (methods section, statistical analysis, paragraph 4) | |
| Results | | | |  | |
| Participants | 13* | (a) Report numbers of individuals at each stage of study—eg numbers potentially eligible, examined for eligibility, confirmed eligible, included in the study, completing follow-up, and analysed | | We provided information on total number of survey participants and those with missing data in the method section and in the S1 Table. | |
|  |  | (b) Give reasons for non-participation at each stage | | N/A | |
|  |  | (c) Consider use of a flow diagram | | N/A | |
| Descriptive data | 14* | (a) Give characteristics of study participants (eg demographic, clinical, social) and information on exposures and potential confounders | | We described characteristics of study participants and their experiences with PC in the results section (paragraphs 1-2, tables 1-4). | |
|  |  | (b) Indicate number of participants with missing data for each variable of interest | | We specified number of participants with missing data in the S1 Table and in the statistical analysis section (paragraph 2) | |
| Outcome data | 15* | Report numbers of outcome events or summary measures | | We reported numbers of outcome and summary measures in the result section (paragraph 1, last sentence) | |
| Main results | 16 | (*a*) Give unadjusted estimates and, if applicable, confounder-adjusted estimates and their precision (eg, 95% confidence interval). Make clear which confounders were adjusted for and why they were included | | We provided data on confounder-adjusted estimates and their precision (eg, 95% confidence interval) (results section). We specified that “several social and health service-related factors are associated with poor SRH. Individual factors linked to lower health status include unhealthy lifestyle and chronic diseases that affect mental and physical health. Although some aspects of the relationship between socio-demographic factors and SRH are still inconclusive, it has been reported that older age, low schooling, low socio-economic status, low social capital and low health insurance coverage are associated with poor SRH Based on survey data availability, we included in the analysis the following covariates: sex, age, schooling, chronic disease, and the type of health insurance. (method section, study variables, paragraphs 4-5). | |
|  |  | (*b*) Report category boundaries when continuous variables were categorized | | N/A | |
|  |  | (*c*) If relevant, consider translating estimates of relative risk into absolute risk for a meaningful time period | | N/A | |
| Other analyses | 17 | Report other analyses done—eg analyses of subgroups and interactions, and sensitivity analyses | | N/A | |
| Discussion | | | |  | |
| Key results | 18 | Summarise key results with reference to study objectives | | On the first paragraph of the discussion section we summarized that “This secondary analysis of a nationally representative survey of the urban population in six LAC countries found that higher overall patient-centered PC experience was associated with excellent and very good SRH. At the same time, specific features of PC associated with excellent and very good SRH differed among countries, with elements from the domains of contact with clinic, communication, technical quality, and coordination showing significant associations in at least one country..” | |
| Limitations | 19 | Discuss limitations of the study, taking into account sources of potential bias or imprecision. Discuss both direction and magnitude of any potential bias | | At the end of the discussion section we specified that “The study has several limitations. First, it is an observational analysis of a cross-sectional survey, which does not allow making causal inferences or identifying the direction of the association between the study variables. Bidirectional relationship can be possible between higher SRH and some healthcare experiences. For instance, from the one hand people with poorer health are less likely to give the clinician credit for solving issues, from the other hand, worse health problems are harder to solve. Second, due to the high prevalence of missing data, the analysis included IP-weighting, therefore we had to assume that the population with a regular source of care is exchangeable conditional on covariates with the population without a regular source of care; if this assumption was violated, the results would not be generalizable to those without a regular source of care. Third, in cross-national comparisons of survey data, cultural differences may lead to different interpretations of the questions being asked of respondents. For this reason, questionnaires had to be adapted for the characteristics of each country. Rather than focusing on the specificities of service provision of each country, this study aimed at identifying the broader roles of primary care that may affect patient experience. Fourth, the results of our study can be generalizable only to urban populations of the analyzed six countries, as the samples were designed to represent national urban populations in each country. The results do not represent experiences of rural populations. Fifth, our findings cannot be generalized to other low-and middle-income countries, because of the different characteristics of their healthcare systems. Finally, information on type of employment and income was not collected by the survey; however, information on the level of education and health insurance was available and included in this study.” | |
| Interpretation | 20 | Give a cautious overall interpretation of results considering objectives, limitations, multiplicity of analyses, results from similar studies, and other relevant evidence | | We gave a cautious overall interpretation of results considering objectives, limitations, multiplicity of analyses, results from similar studies, and other relevant evidence (discussion section, paragraphs 1-4) | |
| Generalisability | 21 | Discuss the generalisability (external validity) of the study results | | At the end of the discussion section we specified that our findings “can be generalizable only to urban populations of the analyzed six countries, as the samples were designed to represent national urban populations in each country. The results do not represent experiences of rural populations. Fifth, our findings cannot be generalized to other low-and middle-income countries, because of the different characteristics of their healthcare systems.” | |
| Other information | | | |  | |
| Funding | 22 | Give the source of funding and the role of the funders for the present study and, if applicable, for the original study on which the present article is based | | We specified that the publication of this work was supported by the Bill & Melinda Gates Foundation (Kruk, OPP1161450). The funders had no role in study design, data collection and analysis, decision to publish, or preparation of the manuscript. | |
