# Supplementary material for: Patient-centered primary care and self-rated health in 6 Latin American and Caribbean countries: Analysis of a public opinion cross-sectional survey
Source: PLoS Med. 2018 Oct 9;15(10):e1002673. doi: 10.1371/journal.pmed.1002673 (PMC6177127; doi:10.1371/journal.pmed.1002673)
Supplement: S1 Table — (DOCX) [file pmed.1002673.s002.docx]

**S1 Table:** **Missing data by country**

|  | **Brazil** | **Colombia** | **El Salvador** | **Jamaica** | **Mexico** | **Panama** |
| --- | --- | --- | --- | --- | --- | --- |
| Original sample, n | 1501 | 1501 | 1500 | 1506 | 1503 | 1501 |
| Missing data, given that lack of access to a regular source of PC, n (%) | 555 (36.9) | 421 (28.0) | 646 (43.1%) | 245 (16.3) | 312 (20.8) | 349 (23.3) |
| Missing data, due to the missing information in the responses to particular questions, n (%) | 72 (4.8) | 71 (4.7) | 42 (2.8) | 121 (8.0) | 9 (0.6) | 69 (4.6) |
| Final sample | 874 | 1009 | 812 | 1140 | 1182 | 1083 |
