# Supplementary material for: Patient-centered primary care and self-rated health in 6 Latin American and Caribbean countries: Analysis of a public opinion cross-sectional survey
Source: PLoS Med. 2018 Oct 9;15(10):e1002673. doi: 10.1371/journal.pmed.1002673 (PMC6177127; doi:10.1371/journal.pmed.1002673)
Supplement: S2 Table — (DOCX) [file pmed.1002673.s003.docx]

**S1 Table: Results of the tests for additive interactions: Relative excess risk due to interaction and 95% CI**

| Patients´ experience with PC | Easy to contact | Never tried to contact | Opportunity to ask questions | Spends enough time with patient | Explains things in a way that is easy to understand | Knows medical history | Advises about healthy lifestyles | Preventive exams up to date | Solves most of health problems | Coordinate care with other physicians or sources of care | Not necessary to coordinate care |
| --- | --- | --- | --- | --- | --- | --- | --- | --- | --- | --- | --- |
| Brazil | **-0.41**  **(-0.78, -0.04)** | -0.01  (-0.63, 0.63) | **0.37 (0.02, 0.71)** | -0.20  (-0.54, 0.14) | 0.16  (-0.21, 0.54) | **-0.70**  **(-1.08, -0.33)** | -0.02  (-0.32, 0.35) | -0.01  (-0.33, 0.31) | 0.04  (-0.32, 0.41) | **-0.54**  **(-0.94,**  **-0.14)** | 0.43  (-1.32, 2.18) |
| Colombia | 0.16  (-0.16, 0.48) | 0.36  (-0.14, 0.85) | 0.22 (-0.18, 0.63) | 0.11  (-0.26, 0.47) | 0.21  (-0.19, 0.60) | 0.20  (-0.16, 0.56) | 0.17  (-0.17, 0.52) | 0.13  (-0.20, 0.46) | 0.36  (-0.02, 0.74) | 0.06  (-0.27, 0.39) | **-0.63**  **(-1.15,**  **-0.10)** |
| El Salvador | 0.22  (-0.09, 0.52) | 0.24  (-0.46, 0.94) | -0.21 (-0.59, 0.17) | -0.17, (-0.50, 0.16) | -0.23  (-0.63, 0.18) | -0.07  (-0.40, 0.27) | 0.18  (-0.11, 0.48) | -0.25  (-0.57, 0.07) | -0.08  (-0.48, 0.31) | 0.18  (-0.13, 0.48) | -0.18  (-1.07, 0.72) |
| Jamaica | -0.07  (-0.47, 0.33) | 0.31  (-1.16, 1.78) | 0.05 (-0.29, 0.39) | -0.04  (-0.36, 0.27) | 0.13  (-0.23, 0.49) | -0.07  (-0.37, 0.25) | -0.22  (-0.55, 0.11) | 0.15  (-0.16, 0.46) | 0.25  (-0.08, 0.58) | -0.02  (-0.34, 0.30) | **0.67**  **(0.10, 1.25)** |
| Mexico | **0.22 (0.04, 0.41)** | -0.30  (-0.63, 0.03) | 0.11 (-0.14, 0.36) | **0.27 (0.07, 0.47)** | **0.34 (0.14, 0.54)** | **0.36 (0.17, 0.55)** | 0.03  (-0.16, 0.22) | -0.13  (-0.32, 0.06) | **0.23 (0.01, 0.45)** | **0.22**  **(0.03, 0.421)** | **0.41**  **(0.04, 0.79)** |
| Panama | -0.12  (-0.41, 0.17) | -0.32  (-1.01, 0.38) | **-0.42 (-0.81, -0.03)** | 0.04  (-0.33, 0.25) | **-0.61**  **(-1.02,**  **-0.19)** | -0.08  (-0.42, 0.26) | -0.09  (-0.37, 0.19) | 0.11  (-0.19, 0.42) | **-0.41**  **(-0.81,**  **-0.01)** | 0.10  (-0.20, 0.40) | -0.26  (-0.86, 0.33) |

Bold values indicate significance at p ≤ 0.05

**Supporting Information 2. Results of the tests for additive interactions: Relative excess risk due to interaction and 95% CI**

|  | **Overall Patient-Centered PC score** |
| --- | --- |
| Brazil | -0.06  (-0.94, 0.082) |
| Colombia | 0.80  (-0.07, 1.68) |
| El Salvador | 0.16  (-0.66, 0.97) |
| Jamaica | 0.74  (-0.03, 01.52) |
| Mexico | **0.55 (0.09, 1.02)** |
| Panama | -0.48  (-1.38, 0.42) |

Bold values indicate significance at p ≤ 0.05
